# A prospectively collected observational study of pelvic floor muscle strength and erectile function using a novel personalized extracorporeal perineometer

**DOI:** 10.1038/s41598-021-97230-6

**Published:** 2021-09-15

**Authors:** Jung Kwon Kim, Young Ju Lee, Hwanik Kim, Sang Hun Song, Seong Jin Jeong, Seok-Soo Byun

**Affiliations:** 1 Department of Urology, Seoul National University College of Medicine, Seoul National University Bundang Hospital, 173-82, Gumi-Ro, Bundang-gu, Seongnam-si, Gyeonggi-do 13620 Korea; 2 Department of Urology, CHA Ilsan Medical Center, Goyang, Korea; 3 Department of Urology, Seoul National University College of Medicine, Seoul, Korea; 4 Department of Medical Device Development, Seoul National University, Seoul, Korea

**Keywords:** Medical research, Urology

## Abstract

To investigate the association between pelvic floor muscle strength and erectile function in a prospectively collected observational cohort. 270 male volunteers were prospectively collected and grouped by International Index of Erectile Function-5 (IIEF-5) scores. Pelvic floor muscle strength was compared. Patients with obvious neurologic deficits, abnormal pelvic bones, history of pelvic radiation therapy, prostatectomy, or urinary incontinence were excluded. We analyzed 247 patients with mean (± standard deviation, SD) age of 62.8 (± 10.1) years. Mean (± SD) maximal and average strength were 2.0 (± 1.5) and 1.1 (± 0.8) kgf, respectively. Mean (± SD) endurance and IIEF-5 scores were 7.2 (± 2.6) seconds and 13.3 (± 7.9), respectively. Patients with IIEF-5 scores ≤ 12 tended to be older, with a higher occurrence of hypertension and lower body mass index. Age [odds ratio (OR) 1.08, 95% confidence interval (CI) 1.04–1.12, *p* < 0.001], and maximal strength < 1.9 kgf (OR 2.62, 95% CI 1.38–4.97, *p* = 0.003) were independent predictors for IIEF-5 scores ≤ 12 in multivariate regression analysis. Patients with erectile dysfunction were older and showed lower pelvic floor muscle maximal strength. Future prospective trials needed for using physiotherapy are required to verify our results.

## Introduction

Behavioral therapy is useful in the treatment of an overactive bladder, as well as stress, urge, and mixed urinary incontinence, nocturia and neurogenic detrusor overactivity^1^. Pelvic floor muscle training (PFMT) is a behavioral therapy commonly used for the conservative management of urinary incontinence^2^. PFMT is offered as first-line conservative therapy for women with stress or urge urinary incontinence. In men, PFMT is used as the primary conservative treatment for urinary incontinence after radical prostatectomy. The previous literature has shown that pelvic floor muscle strength can be associated with urinary incontinence in men similar to the association observed in women^3,4^. This therapy is free of any adverse events and should be offered to all men and women who may benefit from it. Evidence suggests that in addition to its association with urinary incontinence, the pelvic floor musculature shows a close relationship with erectile function. Contractions of pelvic floor muscles, in particular the ischiocavernosus and bulbocavernosus, produce an intracavernous pressure increase and influence penile rigidity. Several studies reported that weak pelvic floor muscles would lead to erectile dysfunction (ED) and pelvic floor muscle exercise is currently regarded as being helpful for ED in men^5–7^.

Pelvic floor muscle strength can be assessed using digital palpation, visual observation, electromyography, manometry, or ultrasonography. Digital assessment is most widely used in clinical practice^1^. External visual observation of the perineum enables the patient and clinician to know what happens during contraction of the pelvic floor muscles^8^. Real-time trans-abdominal and trans-perineal ultrasound can both be utilized to evaluate pelvic floor contractions. Both have shown to be feasible in assessing movement of pelvic structures during contraction^9,10^. However, in men, no standard method has been described to measure perineal muscle function^4^. In the current study, we used a new perineometer to measure the perineal body tone without undressing patients. In addition, we attempted to assess whether pelvic floor muscle strength was related to erectile function in these patients.

## Materials and methods

### Ethics statement

The Institutional Review Boards of Seoul National University Bundang Hospital approved this study (IRB approval number: B-1506/304-003). We obtained written informed consents from all patients who were enrolled. Personal identifiers were completely removed and the data were analyzed anonymously. Our study was conducted according to the ethical standards recommended by the 1964 Declaration of Helsinki and its later amendments.

### Study population

We enrolled 270 men for complaints other than voiding/erectile disorders in this prospective observational study between August 2015 and July 2017. Volunteers included men without any urological disorders with an interest in using the novel perineometer device, as well as patients visiting the clinic for follow-up after medical check-up due to non-related laboratory or imaging abnormalities including PSA elevation, hematuria, renal cyst or mass, and urinary stones. Exclusion criteria included patients with obvious neurological deficits, abnormal pelvic bones, history of pelvic radiation therapy/surgery, prostatectomy, or urinary incontinence. A single study coordinator provided brief instructions to all patients regarding the use of the AnyKegel device (Furun Medica Co., Ltd. South Korea), after which perineometric measurements were performed. Questionnaires assessing the International Prostate Symptom Score (IPSS) and the International Index of Erectile Function-5 (IIEF-5) score were evaluated. Patient demographics including age, body mass index (BMI), history of diabetes mellitus (DM), hypertension, smoking habits, and alcohol consumption were reviewed retrospectively.

### Pelvic perineometric measurements

**Figure 1:**
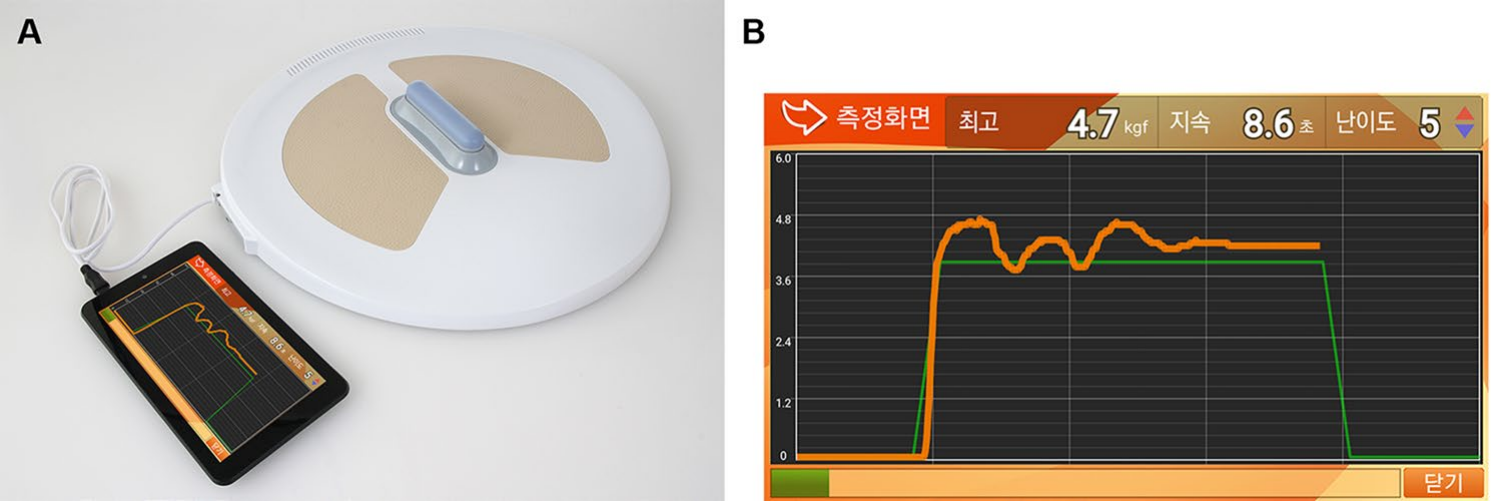
Image shows an extracorporeal perineometer used to measure the pelvic floor muscle strength (AnyKegel). (**A**) The perineometer shows a smart phone-based display. (**B**) Image shows a display of the program during the measurement.

AnyKegel is a portable device with a portable extracorporeal perineometer and a smart phone-based biofeedback program by visualizing the strength of the pelvic floor muscles (Fig. 1). The technique in the device applies the same principle as was previously reported for the management of female stress urinary incontinence^11^. We measured the perineal muscle tone in patients who visited outpatient clinic for complaints other than voiding and/or erectile dysfunction. The sensor was placed near the perineal body. The principle used for measurement was the same as reported in previous report^11^. Details including the patient’s height and weight were entered into the software using a smart phone-based application. The sensor located within the device is elevated to the loosened pelvic floor muscle after confirming entered baseline information including user's height and generates 10 kg of force against the pelvic floor muscles. The longitudinally placed sensor presses against the patient’s perineum between the anus and the penis near the perineal body and measures the contractile force of the pelvic floor muscles. The strength and the duration of contraction of the pelvic floor muscles were displayed on the smart phone as a graph. These data were used to motivate and guide patients via the biofeedback technique. All patients were instructed to voluntarily contract their pelvic floor muscles as best as they could and maintain the contraction for the longest possible duration. After 1 session comprising 10-min exercise, maximal strength (MS), average strength (AS) and endurance were displayed and recorded. MS is defined as the maximal force that a muscle can generate. It is calculated from the difference between the highest and lowest (baseline) strength values (if lowest strength value is 0, then, highest value on curve is just the maximal strength) provided by the equipment software, in kgf. AS is a mean value of the strength curve, provided by the equipment software, in kgf. Endurance in our study is equal to the length of time, in seconds (s), during which the participant could maintain a contraction above 60% of maximum strength, provided by the equipment software^12^.

### Statistical analysis

To investigate the correlation between erectile function and the pelvic floor muscle strength, the best cutoff point for the IIEF-5, to best determine the relationship between erectile function and pelvic floor muscle (PFM) strength in our cohort, was estimated by classifying the maximal strength into 2 groups for all possible scores of the IIEF-5. We calculated the mean intergroup difference in maximal strength. The relationship between the IIEF-5 value and the calculated mean difference value (Supplemental Fig. 1) showed a curvilinear (concave) correlation. Therefore, a second-order polynomial regression model after polynomial regression was fitted to the scatter plot and the best cutoff point of the IIEF-5 was identified as 12.

The cutoff point of maximal strength was estimated using receiver operating characteristic (ROC) curve analysis using categorized variables corresponding to IIEF-5 scores > 12. The best cutoff point of maximal strength to predict weak PFM strength was determined to be 1.9 with 68.3% sensitivity and 47.6% specificity.

All analyses were performed using the SPSS software version 22 (IBM). Cutoff points were estimated using the R program (https://www.R-project.org/, version 3.3.2). Values were expressed as mean ± standard deviation (SD). The Student’s t-test was used to compare continuous variables and the chi-square test for categorical variables. The analysis of variance test was used to compare between 3 categorical variables. The Pearson’s correlation coefficient (r) was used to investigate the correlation between 2 continuous variables. Univariate logistic regression analysis was performed to identify variables with statistical significance. Multivariate logistic regression analysis using an enter method was used. A *P* value < 0.05 was considered statistically significant.

### Consent for publication

All authors have provided consent for publication in this journal.

## Results

We included 247 patients in final analysis. Mean (± SD) age was 62.8 (± 10.1) years. Mean (± SD) BMI was 24.9 (± 10.1) kg/m^2^. Mean (± SD) MS was 2.0 (± 1.5) (range 0.1–9.5) kgf. Mean (± SD) endurance was 7.2 (± 2.6) (range 0.2–12.5) seconds. Mean (± SD) AS was 1.1 (± 0.8) (range 0.0–5.6) kgf. Mean (± SD) IPSS voiding and storage, and the quality of life scores were 6.7 (± 5.2), 5.1 (± 3.6), and 2.5 (± 1.7), respectively.

**Figure 2:**
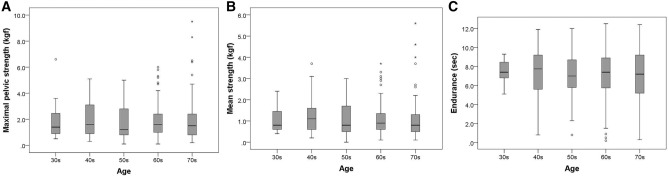
Box-and-whisker plots for maximal pelvic floor muscle strength (**A**), average strength (**B**), and endurance (**C**) based on age. The boxes show the interquartile range (IQR) with the median value expressed as a horizontal line, whiskers show the range of 1.5 × IQR. Circles indicate outliers and the asterisk indicates far outside values.

In regarding perineometer measurements, age was not correlated with MS (r = 0.036), AS (r = -0.005), and endurance (r = -0.036). The distribution of pelvic muscle strength according to the age by decades was displayed in Fig. 2 as a box plot. The IPSS scores (mild [0–7], moderate [8–19] and severe [20–35]) were not associated with maximal strength, average strength, and endurance (Supplemental Table 1).

**Table 1:** Patient characteristics based on the International Index of Erectile Function-5 scores.

	IIEF > 12 (n = 143)	IIEF ≤ 12 (n = 104)	*P*
Age (years)	59.7 ± 10.8	67.1 ± 7.2	< 0.001
BMI (kg/m^2^)	25.2 ± 3.1	24.4 ± 2.4	0.021
IPSS voiding score	5.8 ± 5.1	8.0 ± 5.2	0.001
IPSS storage score	4.5 ± 3.4	6.1 ± 3.6	< 0.001
IPSS QoL	2.3 ± 1.6	2.9 ± 1.8	0.004
DM (n, %)	25 (17.6%)	17 (16.3%)	0.795
Hypertension (n, %)	58 (40.6%)	61 (58.7%)	0.005
**Smoking (n, %)**			
Never	42 (29.4%)	31 (29.8%)	0.080
Current smoker	30 (21.0%)	11 (10.6%)	
Past smoker	71 (49.7%)	62 (59.6%)	
**Alcohol (n, %)**			0.090
Never	61 (43.0%)	53 (51.0%)	
Social	74 (52.1%)	41 (39.4%)	
Heavy	7 (4.9%)	10 (9.6%)	
Endurance (sec)	7.1 ± 2.5	7.2 ± 2.6	0.854
Average strength (kgf)	1.2 ± 0.8	1.0 ± 0.9	0.135
Maximal strength < 1.9 kgf (n, %)	75 (52.4%)	71 (68.3%)	0.013

*BMI* body mass index, *IPSS* international prostate symptom score, *QoL* quality of life, *IIEF* international index of erectile function, *DM* diabetes mellitus, *kgf* kilogram-force.

Table 1 shows patient characteristics based on the IIEF-5 scores. 69 patients had severe ED (IIEF ≤ 7). 124 men and 54 patients had mild-moderate and no ED, respectively. Patients with IIEF-5 scores ≤ 12 tended to be older, showed a lower BMI, a higher IPSS score, and higher occurrence of hypertension than those with IIEF-5 scores > 12. Multivariate logistic regression analysis showed that age (odds ratio [OR] 1.08, 95% confidence interval [CI] 1.04–1.12, *p* < 0.001), and maximal strength < 1.9 kgf (OR 2.62, 95% CI 1.38–4.97, *p* = 0.003) were independent predictors for erectile dysfunction defined as IIEF-5 scores ≤ 12 (Table 2).

**Table 2:** Logistic regression analysis for an International Index of Erectile Function-5 score ≤ 12.

	Univariate logistic regression		Multivariate logistic regression	
	OR (95% CI)	*P*	OR (95% CI)	*P*
Age	1.10 (1.06–1.14)	< 0.001	1.08 (1.04–1.12)	< 0.001
BMI	0.90 (0.81–0.99)	0.023	0.91 (0.81–1.02)	0.090
DM	1.04 (0.54–1.98)	0.912		
Hypertension	2.08 (1.24–3.47)	0.005	1.60 (0.89–2.88)	0.116
Endurance, continuous	1.01 (0.91–1.12)	0.853		
Average strength, continuous	0.76 (0.55–1.04)	0.089		
Maximal strength, < 1.9 kgf	1.95 (1.15–3.31)	0.013	2.62 (1.38–4.97)	0.003

*OR* odds ratio, *BMI* body mass index, *kgf* kilogram-force.

## Discussion

Our study analyzed 247 patients, of whom 104 had IIEF ≤ 12. Men with a cutoff value of IIEF ≤ 12 tended to be older with low pelvic floor muscle maximal strength of < 1.9 kgf. Perineometry is a common technique used to measure and monitor the pelvic floor muscle strength. It is also useful as a biofeedback device and for pelvic floor muscle exercise. Perineometric measurements tend to accurately correspond to those obtained by digital palpation or manual testing^13–15^. Perineometry is a reliable method to measure pelvic floor muscle strength and endurance^16^. Normal parameters related to pelvic floor muscle strength are important for baseline assessment. However, only a few studies have investigated normal parameters^13,17^. The mean values presented in our study can be used as baseline values for future studies for establishing normal range in a healthy population. In addition, current study also showed that pelvic floor muscle strength was not associated with age or voiding symptoms.

Several studies have evaluated pelvic floor muscle contractions in women using a perineometer; however, very few studies have used a perineometer in men. Rigatti et al.^4^ used the Beco perineometer to measure the perineal body tone. Their study was the first to provide evidence regarding an objective measurement of the pelvic floor muscle strength and postoperative recovery of continence in men. They measured the pelvic floor muscle strength in a supine position. An anal perineometer, which resembles the vaginal probes used in women, has also been used in men^3,18^. Although the aforementioned methods are useful, a disadvantage is that they require at least partial undressing, which can be not only embarrassment but also inconvenience and unnecessarily time-consuming for patients in real-world clinical practice. In contrast, our novel extracorporeal biofeedback device can be used without undressing and can provide biofeedback with daily use at any convenient location.

The association between pelvic floor muscle strength and erectile function is explained by the activation of the superficial bulbocavernosus and the ischiocavernosus muscles during penile erection^19^. During erection, the penis functions as a blood-filled closed chamber. The activity of these muscles increases the intracavernosal pressure to retain blood within the penis to maintain the erection^7,20^. The bulbocavernosus muscle contracts synchronously with the external anal sphincter^21^, and repeated successive stimulation of the external anal sphincter can elicit an ischiocavernosus muscle response^22^. Therefore, pelvic floor muscle exercise causes contraction of the external anal sphincter, which consequently causes contraction of the aforementioned muscles to enhance erectile function. Dorey et al.^6,23^ have demonstrated that pelvic floor muscle exercise and biofeedback are effective strategies for patients with erectile dysfunction. Our results showed that erectile dysfunction is associated with decreased pelvic floor muscle strength, which reiterates the role of pelvic floor muscle exercise in such patients. Age, obesity, hypertension, and lower urinary tract symptoms are known risk factors for erectile dysfunction^24^. Univariate analysis performed in current study showed a correlation between these known risk factors and erectile dysfunction. However, multivariate analysis showed that only age and lower pelvic floor muscle maximal strength were significant predictors of erectile dysfunction. Erectile dysfunction is often an early symptom/marker of systemic vascular disease^25^, and several conditions affecting vascular health can cause erectile dysfunction. Notably, current study cohort did not include patients presenting for alleged erectile dysfunction. This might have influenced the results of the multivariate analysis. Consequently, we could represent that reduced pelvic floor muscle strength could be an indicator of erectile dysfunction.

The current study has some limitations. First, we could not recruit enough number of younger volunteers due to the relatively old-aged nature of the urologic patient group, in which more than 70% of patients are over 45. In addition, unlike previous studies that have reported measurements in pressure units, we used the kilogram-force unit, which was not comparable with previous studies. In addition, we could not guarantee the proper PFM contraction due to the absence of modalities such as ultrasound to assess the PFM integrity. Thus, the objective morphological assessment of PFM is still needed to increase success rates of PFMT. Even with these limitations, to our best knowledge, this is the first study to report assessment of the pelvic floor muscle strength in men and also the first to utilize a novel extracorporeal device for measurement.

## Conclusions

Patients with severe-to-moderate erectile dysfunction (defined as IIEF-5 scores ≤ 12) tend to be older and show lower pelvic floor muscle maximal strength. However, pelvic floor muscle strength was not associated with age. Strategies to increase the maximal strength including physiotherapy or biofeedback might be helpful to improve erectile function. Future studies focusing on physiotherapy to treat erectile disorders are necessary to confirm the relationship between erectile function and pelvic floor muscle strength.

## Supplementary Information

Supplementary Information.

## Data Availability

The authors confirm that the data supporting the findings of this study are available within the article [and/or] its supplementary materials.
