# Supplementary material for: A prospectively collected observational study of pelvic floor muscle strength and erectile function using a novel personalized extracorporeal perineometer
Source: Sci Rep. 2021 Sep 15;11:18389. doi: 10.1038/s41598-021-97230-6 (PMC8443575; doi:10.1038/s41598-021-97230-6)
Supplement: Supplementary file 1 — Supplementary Information. [file 41598_2021_97230_MOESM1_ESM.pdf]

Title Page:

TITLE

A prospectively collected observational study of pelvic floor muscle strength and erectile function using a novel personalized extracorporeal perineometer

AUTHORS AND INSTITUTIONS

Jung Kwon Kim[1,+], Young Ju Lee[2,+], Hwanik Kim[1], Sang Hun Song[1], Seong Jin Jeong[1,3], Seok-Soo Byun[1,3]*

[1]Department of Urology, Seoul National University Bundang Hospital, Seongnam, Korea

[2]Department of Urology, CHA Ilsan Medical Center, Goyang, Korea

[3]Department of Urology, Seoul National University College of Medicine, Seoul, Korea

[+]Jung Kwon Kim and [+] Young Ju Lee contributed equally to this work.

Corresponding author

Seok-Soo Byun, M.D. PhD.

Professor, Department of Urology, Seoul National University College of Medicine, Seoul National University Bundang Hospital, Seongnam, Korea

173-82, Gumi-Ro, Bundang-gu, Seongnam-si, Gyeonggi-do, 13620, Korea

Tel: 82-31-787-7342

Fax: 82-31-787-4057

E-mail: ssbyun@snubh.org

Supplemental Figure 1. The relationship between the IIEF-5 value and the calculated mean difference in maximal strength.

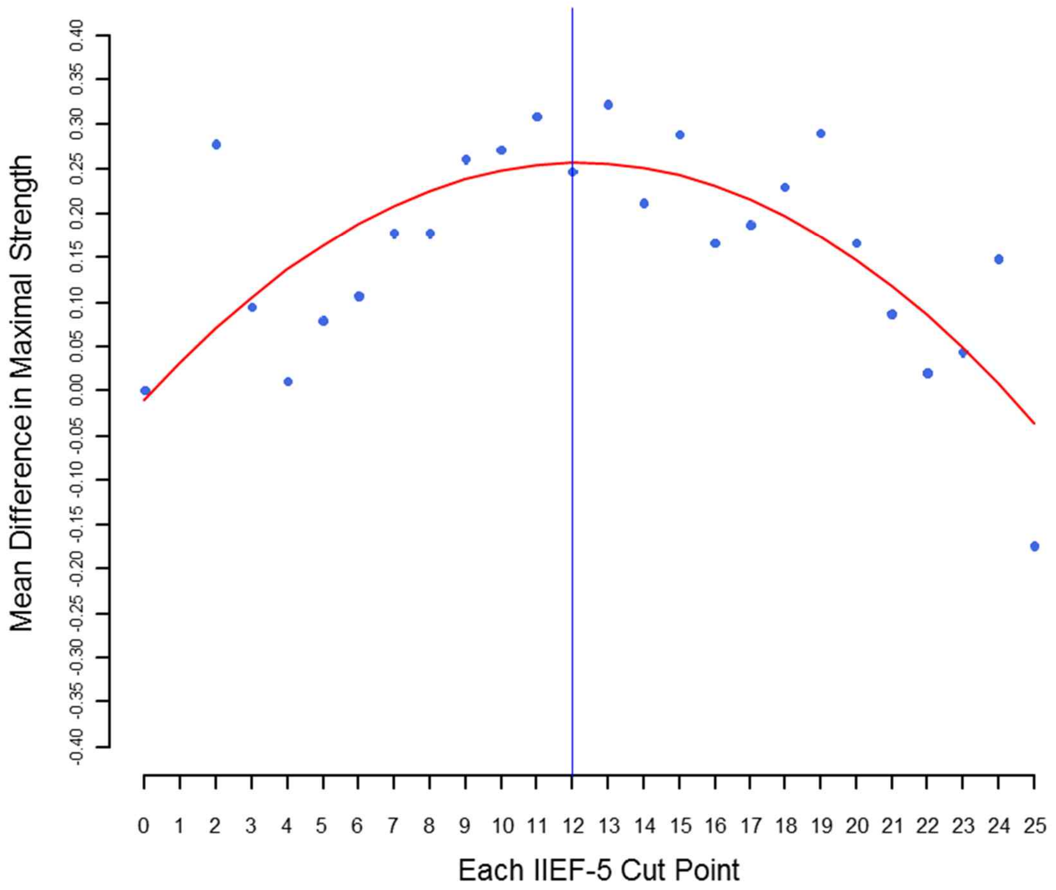

Supplemental Table 1. Pelvic floor muscle strength based on the severity of voiding symptoms

	IPSS mild (0-7) (n=97)	IPSS moderate (8-19) (n=107)	IPSS severe (20-35) (n=48)	P value
Maximal strength (N)	18.1±12.2	18.3±13.8	22.3±20.6	0.240
Mean strength (N)	10.3±6.8	10.4±7.6	12.0±11.0	0.463
Endurance (s)	6.91±2.22	7.33±2.59	7.17±3.08	0.520

IPSS, International Prostate Symptom Score; N, Newton
